# The Dynamics of Prosthetically Elicited Vestibulo-Ocular Reflex Function Across Frequency and Context in the Rhesus Monkey

**DOI:** 10.3389/fnins.2018.00088

**Published:** 2018-05-15

**Authors:** James O. Phillips, Leo Ling, Amy L. Nowack, Christopher M. Phillips, Kaibao Nie, Jay T. Rubinstein

**Affiliations:** ^1^Otolaryngology–Head and Neck Surgery, University of Washington, Seattle, WA, United States; ^2^Washington National Primate Research Center, University of Washington, Seattle, WA, United States; ^3^Virginia Merril Bloedel Hearing Research Center, University of Washington, Seattle, WA, United States; ^4^Epidemiology, University of Washington, Seattle, WA, United States; ^5^Bioengineering, University of Washington, Seattle, WA, United States

**Keywords:** vestibular, prosthesis, vestibulo-ocular reflex, dynamics, eye position, head position

## Abstract

Electrical vestibular neurostimulation may be a viable tool for modulating vestibular afferent input to restore vestibular function following injury or disease. To do this, such stimulators must provide afferent input that can be readily interpreted by the central nervous system to accurately represent head motion to drive reflexive behavior. Since vestibular afferents have different galvanic sensitivity, and different natural sensitivities to head rotational velocity and acceleration, and electrical stimulation produces aphysiological synchronous activation of multiple afferents, it is difficult to assign a priori an appropriate transformation between head velocity and acceleration and the properties of the electrical stimulus used to drive vestibular reflex function, i.e., biphasic pulse rate or pulse current amplitude. In order to empirically explore the nature of the transformation between vestibular prosthetic stimulation and vestibular reflex behavior, in Rhesus macaque monkeys we parametrically varied the pulse rate and current amplitude of constant rate and current amplitude pulse trains, and the modulation frequency of sinusoidally modulated pulse trains that were pulse frequency modulated (FM) or current amplitude modulated (AM). In addition, we examined the effects of differential eye position and head position on the observed eye movement responses. We conclude that there is a strong and idiosyncratic, from canal to canal, effect of modulation frequency on the observed eye velocities that are elicited by stimulation. In addition, there is a strong effect of initial eye position and initial head position on the observed responses. These are superimposed on the relationships between pulse frequency or current amplitude and eye velocity that have been shown previously.

## Introduction

The semicircular canals transduce head rotation to modulate afferent inputs to the vestibular brainstem and cerebellum. This transformation has been modeled as a simple torsion pendulum, which, over a range of frequencies, provides neural representations of head velocity and acceleration to drive a fully compensatory vestibular ocular reflex (VOR), among other behaviors. Indeed, this system is remarkably accurate, providing relatively constant high gain VOR across a range of frequencies up to 20 Hz in rhesus monkeys (Ramachandran and Lisberger, 2005). However, the relative simplicity of this first approximation model masks an extremely complex set of central and peripheral neural elements and physiological processes which work in combination to perform the job of creating a reliable behavioral response from a range of inputs. These complex mechanisms have been largely elucidated in animal models, and much of this work was performed in rhesus monkeys, which have similar anatomy and behavior to humans.

Recently, several laboratories have performed experiments to understand the optimal strategies to electrically stimulate the afferent fibers of the semicircular canals to produce the high consistent gains in response that define the fully compensatory VOR. The purpose of these research efforts is ultimately to develop a working vestibular prosthesis to convert motion information, sensed with a rotational transducer, into electrical stimulation to activate preserved afferent fibers following vestibular hair cell loss, restoring natural behavior (e.g., Thompson et al., 2016). Several excellent recent reviews provide a selective introduction to this literature (e.g., Fridman and Della Santina, 2012; Guyot et al., 2016; Lewis, 2016). Typically, the dependent measure of effective vestibular function is slow phase eye velocity, elicited as an electrical vestibuloocular reflex either in response to real motion or in response to fictive motion due to electrical stimulation alone. We have previously reported longitudinal slow phase eye movement data using brief 2 s constant current amplitude and constant pulse rate stimulation in several monkeys (Phillips et al., 2014, 2016) and in human subjects (Phillips et al., 2013, 2015). In this paper, we examine the relationship between the parameters of biphasic pulse stimulation with a vestibular neurostimulator and the resulting electrically elicited VOR (eVOR) in rhesus monkeys. We do so for our previously reported measure of 2 s stimulations, and across a broad range of physiologically reasonable modulation frequencies, which are known to produce consistent high gain responses to natural rotational stimulation in these animals. In addition, we vary the context of the electrical stimulation by changing the starting eye orbital position and head orientation during stimulation to examine the extent to which the gain of the VOR in monkeys is maintained in response to electrical stimulation in physiologically relevant situations.

## Materials and methods

The experiments described in this paper strictly followed the recommendations of the Society for Neuroscience and the National Research Council (1997, 2003). They exceeded the recommendations of the Association for Assessment and Accreditation of Laboratory Animal Care International and the Institute of Laboratory Animal Resources. All procedures were approved by the Institutional Animal Care and Use Committee of the University of Washington (original PHS assurance number, D16-00292 and current DHS assurance number A3463-01, last 3 year approval date 11/16/2015).

### Device and surgery

**Figure 1:**
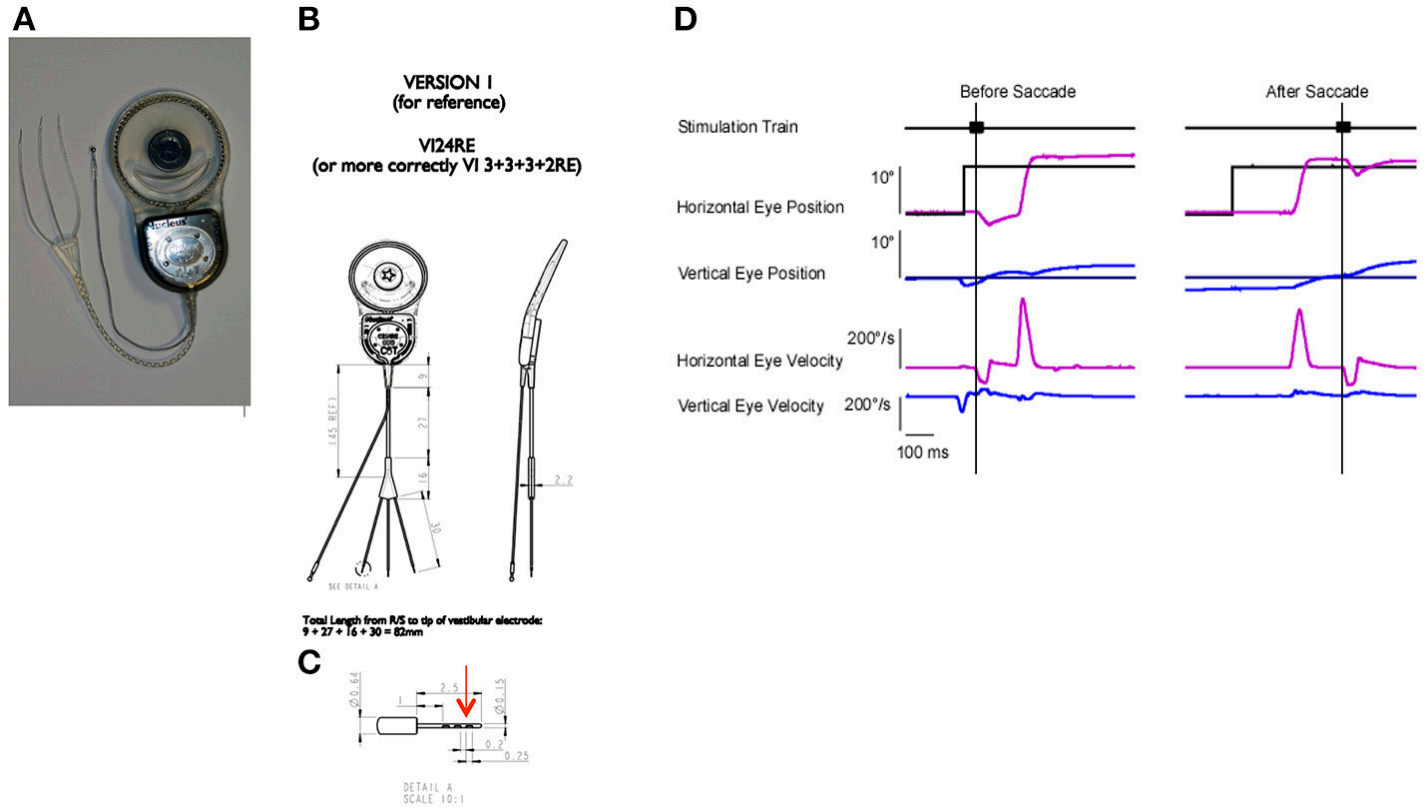
Device and paradigm: **(A)** A photograph of the neurostimulator used in the study. The device has a processor and stimulator attached to a ring shaped radio frequency (RF) link. There is a trifurcated electrode array, with each electrode having a small tip for insertion. Each tip has 3 stimulation sites. **(B)** Device dimensions and design. **(C)** Tip dimensions and design. Red arrow denotes the distal stimulation site. **(D)** Paradigm for stimulus presentation. Animals tracked a point target, which was stepped to different locations. The stimulation was triggered either on the target step (before saccade) or on the resulting saccade (after saccade). The target was eliminated for 500 ms after the stimulation onset. Traces, from top to bottom are the 50 ms stimulation train, horizontal eye position (light blue) and horizontal target position (black), vertical eye position (dark blue), and vertical target position (black), horizontal eye velocity (light blue), vertical eye velocity (dark blue). Vertical lines denote electrically elicited eye movement onset.

Rhesus macaque monkeys were implanted in the right ear with a vestibular neural stimulator based on a cochlear implant (Nucleus Freedom, Cochlear Ltd., Sydney). The design of the device was identical to the devices approved for use in human subjects in a clinical trial for the treatment of Meniere's disease (Golub et al., 2014). The surgical approach has been described previously (Rubinstein et al., 2012) but briefly the device (Figures 1A,B) was placed subcutaneously on the right temporal aspect of the skull. It was oriented so that an RF link, which allowed transdermal external communication with a processor, extended rostrally, while the stimulation and remote ground leads of the device extended caudally. The temporal bone was drilled to expose the 3 semicircular canals, taking care to avoid the facial nerve, which was also visualized. A small fenestration was made in the bone surrounding each semicircular canal adjacent to the ampulla of the canal. The small tip of a single stimulation lead (Figure 1C), containing 3 serially arranged independent stimulation sites, was inserted into the fenestration but parallel to the course of the canal, into the potential space of the perilymphatic compartment with the intention of maintaining the patency of the endolymphatic compartment and the natural rotational response of the canal.

To assure that the electrodes were optimally placed, the device was activated in surgery, and biphasic electrical pulses were used to drive compound action potentials, which were recorded by the device at an adjacent electrode site (Nie et al., 2011; Phillips et al., 2012). Briefly, standard neural response telemetry (NRT) was used to record the vestibular electrical evoked compound action potentials (vECAPs) using a forward masking stimulus to reduce recording artifacts. The amplitude of the N1-P1 response was measured using Nucleus Freedom Custom Sound EP software (v1.3, Cochlear Ltd.). The position of the electrode was changed if such stimulation failed to elicit robust N1-P1 amplitude. The fenestration was then sealed with fascia, and each lead was secured with a stitch of non-absorbable suture. A remote ground was also positioned under the temporalis muscle, although the case of the neurostimulator served as another ground.

In a separate sterile surgery we implanted small restraining lugs to hold the head stationary with respect to the seated monkeys' chair, a scleral eye coil for eye position recording, and a chamber for future brainstem neural recording. The restraining lugs were preformed from dental acrylic, and were attached to the skull of the monkey at 2–3 locations with dental acrylic and small screws. The small stainless steel recording chamber, which was filled with silastic to limit infection, was placed steriotaxically following a craniotomy, and secured in location with small screws and dental acrylic. A preformed scleral coil fabricated from Teflon coated multistranded stainless steel wire was placed in the left eye of each monkey following the method of Judge (Judge et al., 1980). The leads from the coil were led through the posterolateral aspect of the orbit and then subcutaneously to the front stabilization lug, which contained a small electrical connector.

For the purpose of the experiments described here, the vestibular neurostimulator was connected via the RF link to an external processor (NIC-2, Cochlear Ltd., or Nucleus Freedom Speech Processor), which was then connected to a PC computer by means of a USB cable or to a research stimulator (Nuclear Chicago, Chicago, IL) via a buffer amplifier with direct input to the speech processor. The computer ran custom software to deliver instructions to the external processor which in turn instructed the receiver stimulator to deliver predetermined electrical stimuli to individual stimulation sites of the device. The research stimulator could be triggered in real time to deliver a square wave stimulus to drive preprogrammed trains of stimulation pulses. Therefore, in these experiments, the electrical stimulation was substituted for real time modulated activation of the external processor based on head motion.

All experiments were conducted in a sound proof and light tight booth with the monkey sitting in a primate chair that was embedded in a servo-controlled multiaxis rotator (Actek, Seattle, WA). The rotator contained a cylindrical projection screen that moved with the primate chair along with a 2D laser mirror galvanometer system to deliver visual stimuli. The animal was rewarded with applesauce for placing its eye within a settable reward window (typically ±2°) centered on the illuminated spot for a minimum of 1 s. In these experiments, the geometry between the visual stimulus and the monkey's chair and head remained fixed in all conditions.

All stimulation was delivered in complete darkness with the animal's chair and head stationary. The stimuli utilized in these experiments consisted of trains of biphasic pulse stimuli (100 μs per phase and 8 μs gap) delivered to individual semicircular canal electrodes of the right ear through the most distal stimulation site on a given electrode array (Figure 1C, red arrow). We parametrically varied the stimulation current amplitude or pulse frequency to observe the effects on the electrically elicited slow phase eye movements of the vestibulo-ocular reflex.

In Experiment 1, we defined the relationship between stimulation current or pulse frequency and slow phase eye velocity with pseudorandomly delivered 2 s trains of constant current and constant pulse frequency stimuli. The brief nature of the stimulation was selected to reduce adaptation to the repeated stimulation over the course of a recording session. Similar stimuli have been used in previous experiments in our laboratory (Phillips et al., 2014). Trials were initiated while the animal fixated the spot in primary orbital position; i.e., straight ahead. Approximately 100 ms prior to the onset of stimulation, the spot was extinguished.

In experiment 2, we documented the frequency dependence of the eVOR. Two pulse frequency and current amplitude combination pairs were selected from relationships established from previous recording of 2 s trains in experiment 1. These pairs were selected to produce both moderate and low (just at threshold) slow phase eye velocities in the plane of the stimulated canal. During the recoding session, we repeated the stimulation with constant pulse frequency and constant current amplitude 2 s trains, but randomly interleaved these with longer duration trains of current amplitude modulated (AM) or pulse frequency modulated (FM) stimulation, which was sinusoidally varied between the current amplitude and pulse frequency “limits” defined by the stimulation pairs from our 2 s stimulation trains. This way we could examine both the DC response of the VOR to electrical stimulation with 2 s trains, and the AM and FM response of the VOR to comparable stimuli at different modulation frequencies.

To document the context dependence of the response to electrical stimulation, we repeated constant current and constant pulse frequency stimulation trains in two different contexts; i.e., movements initiated from different eye positions and movements initiated in different head orientations.

In Experiment 3, to evaluate the eye position effects on slow phase eye velocity, we initiated electrical stimulation with very brief (50 ms) trains of constant current amplitude and constant pulse frequency stimulation with the eye in different starting positions along the horizontal or vertical meridian. Because we did not know the potential effect of the presence of a preceding visual target on the observed elicited slow phase eye velocities, we initiated stimulation either before or after a saccade to a target step (Figure 1D). In the before saccade condition, the eye was fixating a previous target location after a target step when the stimulation was initiated. In the after saccade condition, the eye was fixating the new target location. For all stimulation, the target was switched off 100 ms prior to stimulus onset and remained off for 500 ms. Target locations were pseudo-randomly varied. In these experiments, therefore, we examined the ability of the vestibular system to respond consistently to a fictive rotational stimulus with the eye in different orbital positions.

In Experiment 4, we initiated all electrical stimulation from primary orbital position. The stimulations were 2 s trains constant current amplitude and constant pulse frequency stimulation in the dark, as in our initial experiments. However, to evaluate head orientation effects, before each block of stimulation we pseudo-randomly varied the orientation of the monkey's head and trunk, by rotating the animal en-bloc into different static roll and pitch tilt orientations prior to stimulation. In so doing, we examined the ability of the vestibular system to respond consistently to a fictive rotational stimulus in different gravitational contexts.

### Data recording and analysis

Eye position data was acquired using a Robinson coil system attached to the multi-axis rotator (CNC Engineering, Seattle, WA). The driver coils maintained a constant orientation with respect to the head of the monkey, the monkey chair, and the visual stimulus because they were mechanically coupled. Eye position, chair position, target position, and target illumination (laser on-off) were digitally sampled at 1 KHz using custom software written in Spike2 (CED, Cambridge, UK). In addition, stimulus pulses reported by the neurostimulator or stimulus artifact recorded from surface electrodes, were sampled at 20 KHz.

Analysis of the data was conducted offline using additional custom software written in Spike2 and Matlab (Mathworks, Natick, MA). Eye position records were marked to define to onset and offset of stimulation. In addition, comparable epochs without stimulation were analyzed for each animal for each test session. The purpose of these later epochs was to quantify any unstimulated drift in eye position in the dark present during the recordings. The eye position records were then digitally differentiated. Based on a settable velocity criterion, the records were desaccaded to eliminate saccades or nystagmus fast phases, and the timing of the fast phases was determined. The different slow phase eye velocity epochs were then subjected to different analyses based on the condition and stimulus type. For control epochs of constant pulse frequency and constant current amplitude stimulation the slow phase eye velocity of each slow phase was subjected to a linear regression using a least-squares method to calculate the average velocity of each resultant slow-phase. We then created a time weighted average of all of the horizontal and vertical eye velocities associated with multiple trials of the same condition. The average of these values was used as a measure of the slow-phase velocity for that condition. The values of slow phase eye velocity associated any spontaneous drift during that test session were subsequently subtracted from the calculated average velocity of the slow phases from stimulated epochs from the same session to provide an measure of the slow phase velocity actually produced by electrical stimulation.

For sinusoidally modulated electrical stimulation pulse trains, a different analysis was performed. Eye position records during sinusoidal stimulation were digitally differentiated and desaccaded, as described previously. A least-squares fit to a sinusoid at the frequency of the stimulus modulation was applied to the eye velocity data to calculate a phase, amplitude, and offset of the sinusoidal eye velocity elicited from the electrical stimulation. The phase was calculated relative to the half amplitude midpoint of the sinusoidal electrical stimulation waveform. For example, if stimulation pulse frequency was modulated between 50 and 250 pps at a constant current amplitude, the half amplitude midpoint would be 150 pps. In addition, a secondary analysis was performed on the data, where the individual cycles of data during electrical stimulation were accumulated into a single composite cycle, which was then fit using a least square approximation to a sinusoid. This was done to check the accuracy of the fits. The results of the first method are reported here.

For all experiments, statistical analyses were performed using a *post-hoc* ANOVA or linear regression models in Statview (SAS Institute, Cary, NC). Statistical significance was at a level of *P* = 0.05.

## Results

Four rhesus macaques and 8 semicircular canal responses were included in this study (Table 1). However, only a subset of the 8 canals was studied in each experiment. For the determination of the velocity of eye movements elicited by constant pulse frequency and constant current amplitude stimulation initiated from primary eye position (Experiment 1), all animals and all canals were used. Longitudinal data comparable to the data of experiment 1 have been reported earlier (Phillips et al., 2014). For the determination of the relationship between modulation frequency and slow phase velocity during sinusoidal stimulation (Experiment 2), data from 4 monkeys and 6 stimulated canals were obtained. 4 of the canals were lateral canals, and there was one posterior canal and one anterior canal stimulated as well. For the data examining the relationship between eye starting position and eye slow phase velocity (Experiment 3), data from 2 monkeys and 4 canals were obtained. Each monkey contributed data from lateral canal and posterior canal stimulation. Finally, for the data examining the relationship between head orientation and slow phase velocity (Experiment 4), data from one monkey and all 3 canals (anterior, posterior, and lateral) were obtained.

**Table 1:** Canals and animals used in each experiment.

**Canal**	**Monkey**	**Type**	**Exp1**	**Exp 2**	**Exp 3**	**Exp 4**
MC1	M1	Lateral	X	X		
MC2	M2	Anterior	X	X		X
MC3	M2	Posterior	X	X		X
MC4	M2	Lateral	X	X		X
MC5	M3	Lateral	X	X	X	
MC6	M4	Lateral	X	X	X	
MC7	M3	Posterior	X		X	
MC8	M4	Posterior	X		X	

*Canal, Monkey, and Type correspond to the canal # used in the text, the corresponding animal for that canal, and the location of the canal in the right ear. X denotes that that canal was used in the listed experiment (Exp#)*.

### Experiment 1 (2 s constant parameter biphasic pulse stimulation)

**Figure 2:**
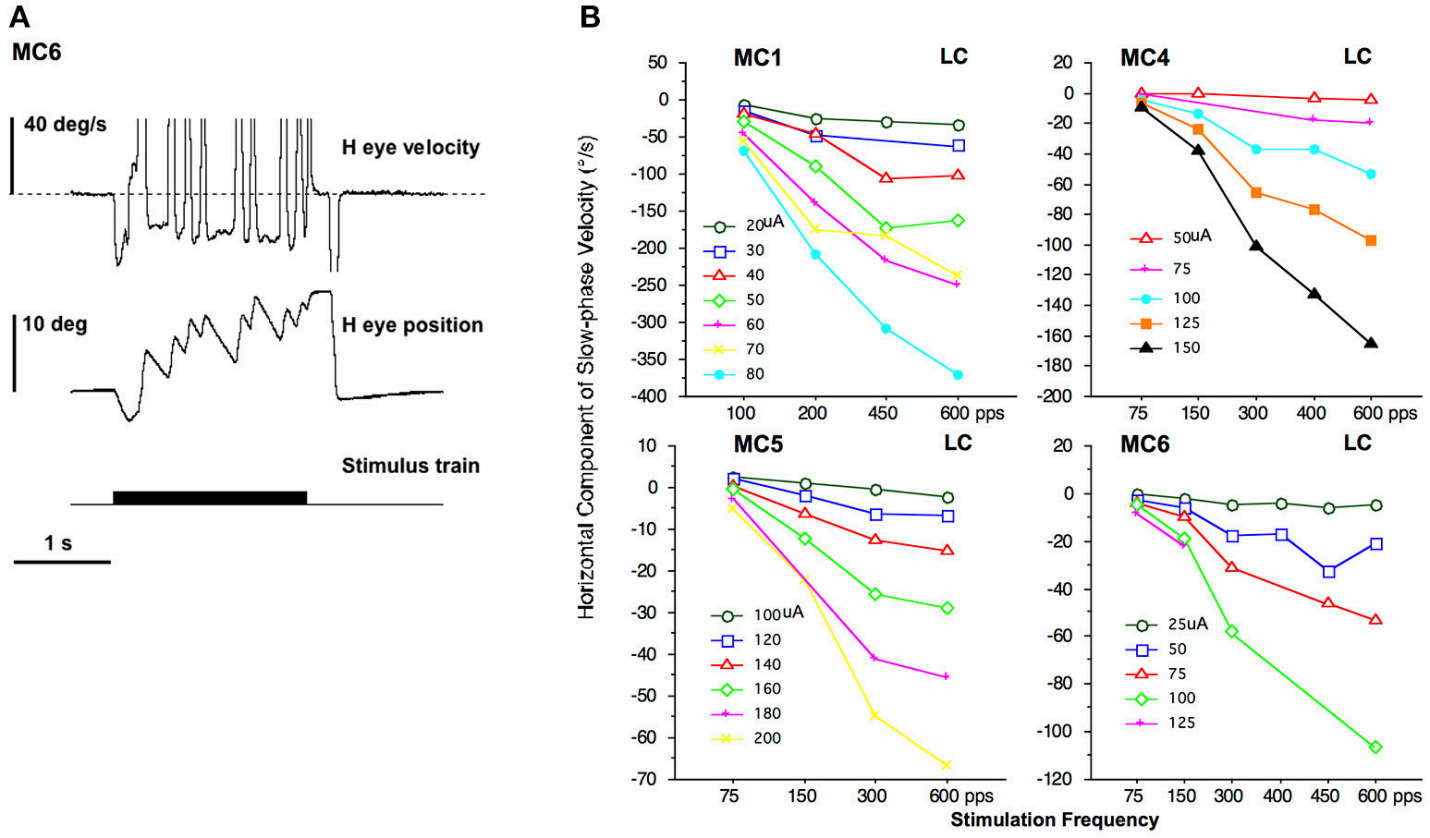
2 s constant pulse frequency and constant current amplitude (constant parameter) biphasic pulse train stimulation at the distal electrode of a canal array elicits nystagmus. **(A)** Representative response to 2 s stimulation in the right lateral canal (MC6) of monkey M4. Traces from top to bottom are horizontal eye velocity, horizontal eye position, and stimulus train. The horizontal and vertical eye position are at primary position (straight ahead) at the onset of stimulation. The stimulation parameters are biphasic pulses 125 μA, 300 pps, 100 μs per phase, 8 μs gap. **(B)** Average slow phase velocity of nystagmus elicited by 2 s constant parameter stimulation trains at different pulse frequencies and current amplitudes for 4 right lateral canals (MC1, MC4, MC5, and MC6). Each point represents average data from multiple trials and slow phases. Each line represents data at a different current amplitude. Negative velocity values are leftward. Canal orientation for each canal is indicated as LC (lateral canal).

In all monkeys we performed electrical stimulation with 2 s trains of constant current amplitude and constant pulse frequency stimulation. These stimuli elicited a constant velocity slow phase nystagmus largely in the plane of the implanted semicircular canal. Figure 2A shows the result of electrical stimulation of the right lateral canal in monkey M4 (canal MC6). A right beating constant slow phase velocity nystagmus was elicited by 2 s stimulation at 125 μA with a pulse rate of 300 pps. In Figure 2A, it appears as though there is a progression in eye position throughout the stimulation, producing a net deviation of the eye from 2 s electrical stimulation. However, this was idiosyncratic to this representative trial. There was, in fact, no statistically significant (*P* > 0.05) difference in the mean beginning and end position over all trials of 2 s stimulation in any animal. Furthermore, there was no significant difference in mean beginning and ending vertical eye position between anterior canal stimulation and posterior canal stimulation in the same animal, despite the fact that the slow phase vertical eye velocities were in different directions (not shown in Figure 2). These data suggest that constant frequency and constant current amplitude stimulation produces a constant velocity rotational input to the central nervous system.

Changing the stimulation parameters produces changes to the observed slow phase eye velocity of the elicited nystagmus. Figure 2B plots the slow phase velocity data from the four right lateral canals of monkeys M1–M4 (MC1, MC4, MC5, and MC6, respectively). With increasing current amplitude (separate lines) or increasing pulse frequency there is an increase in the leftward (negative) slow phase velocity of the observed nystagmus, in addition to a small vertical eye velocity component (not shown). Indeed, the slow phases in response to electrical stimulation in non-human primates can increase to very high velocities (see Figure 2B), which are distinguishable from fast phases only by their direction, which is assumed to be comparable to that at lower stimulation currents and frequencies. These measures therefore provide a mapping of stimulation current to rotational velocity, at least for constant parameter stimulus trains.

**Figure 3:**
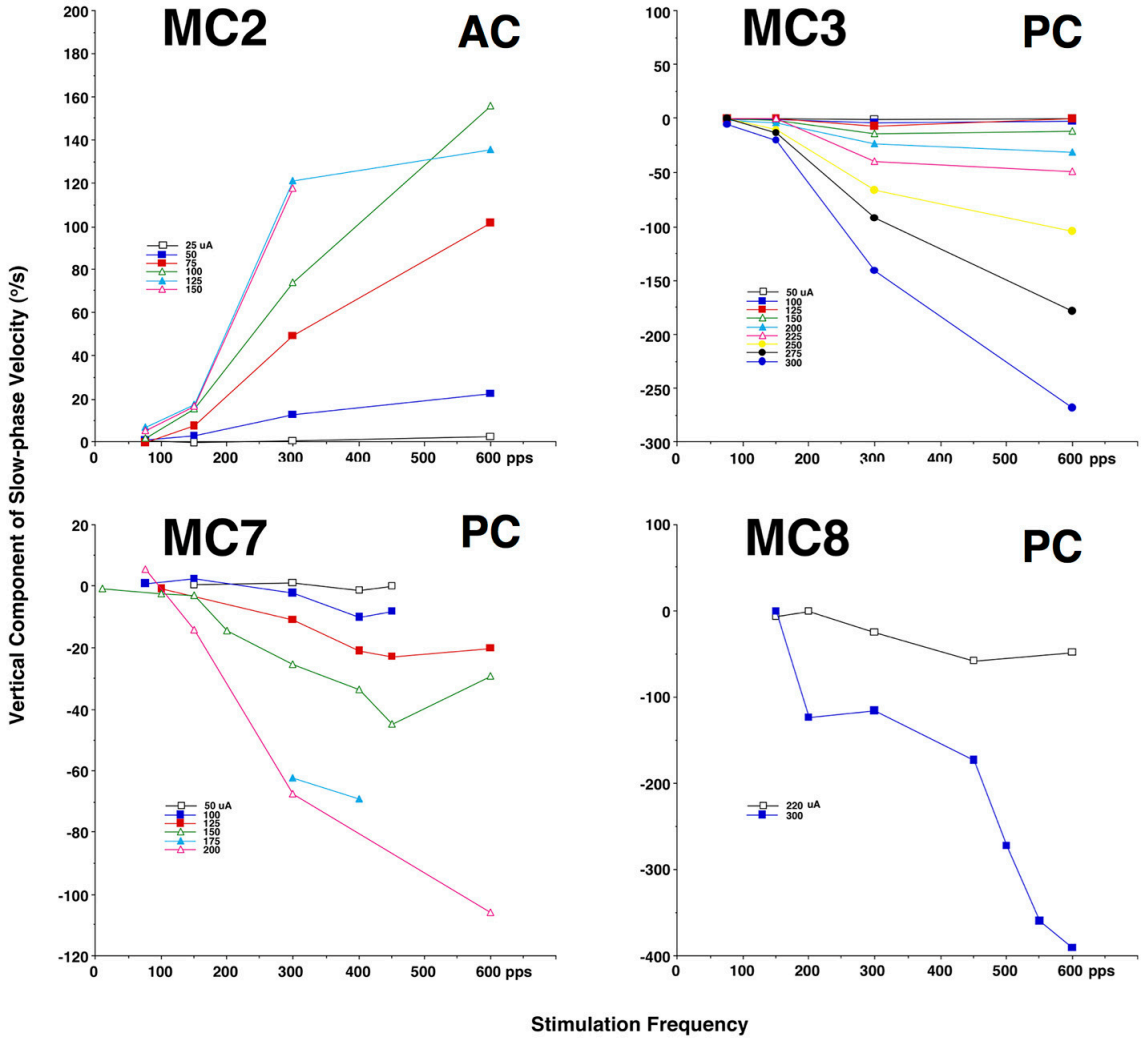
Average slow phase velocity of nystagmus elicited by 2 s constant parameter stimulation trains at different pulse frequencies and current amplitudes for 1 right anterior canal (MC2) and 3 right posterior canals (MC43, MC7, and MC8). Each point represents average data from multiple trials and slow phases. Each line represents data at a different current amplitude. Negative velocity values are downward. Canal orientation for each canal is indicated as AC (anterior canal), or PC (posterior canal).

A comparable experiment was also performed for the vertical canals. Figure 3 plots the slow phase velocity data from four vertical canals of monkeys M2–M4 (MC2, MC3, MC7, MC8). For the one anterior canal (AC) that was stimulated (MC2), increasing current amplitude or increasing pulse frequency produced increasing upward eye velocity, whereas for the three posterior canals (PC) that were stimulated (MC3, MC7, MC8), there was an increase in downward eye velocity with increasing current amplitude or increasing pulse frequency. In the case of the vertical canals, very small horizontal eye velocity was also observed (not shown). It is important to note that since the monkeys used in these experiments were implanted with 2 dimensional eye coils, torsional eye movements were not recorded here.

### Experiment 2 (AM and FM biphasic pulse stimulation)

Since we know that the sensitivity of vestibular afferents is related both to head velocity and acceleration, it may be the case that providing a sinusoidal time varying electrical stimulation train produces a response that differs substantially from the short 2 s constant parameter stimulation trains shown above. To evaluate this, we chose stimulation parameters that evoked moderate slow phase eye velocities, and also stimulation parameters that evoked little or no slow phase eye velocity from each canal to be tested. We then sinusoidally modulated either the stimulation current amplitude or the stimulation pulse frequency between these limits, while holding the other parameter constant; i.e., stimulation pulse frequency or current amplitude, respectively. Trials at different sinusoidal modulation frequencies were presented pseudorandomly.

**Figure 4:**
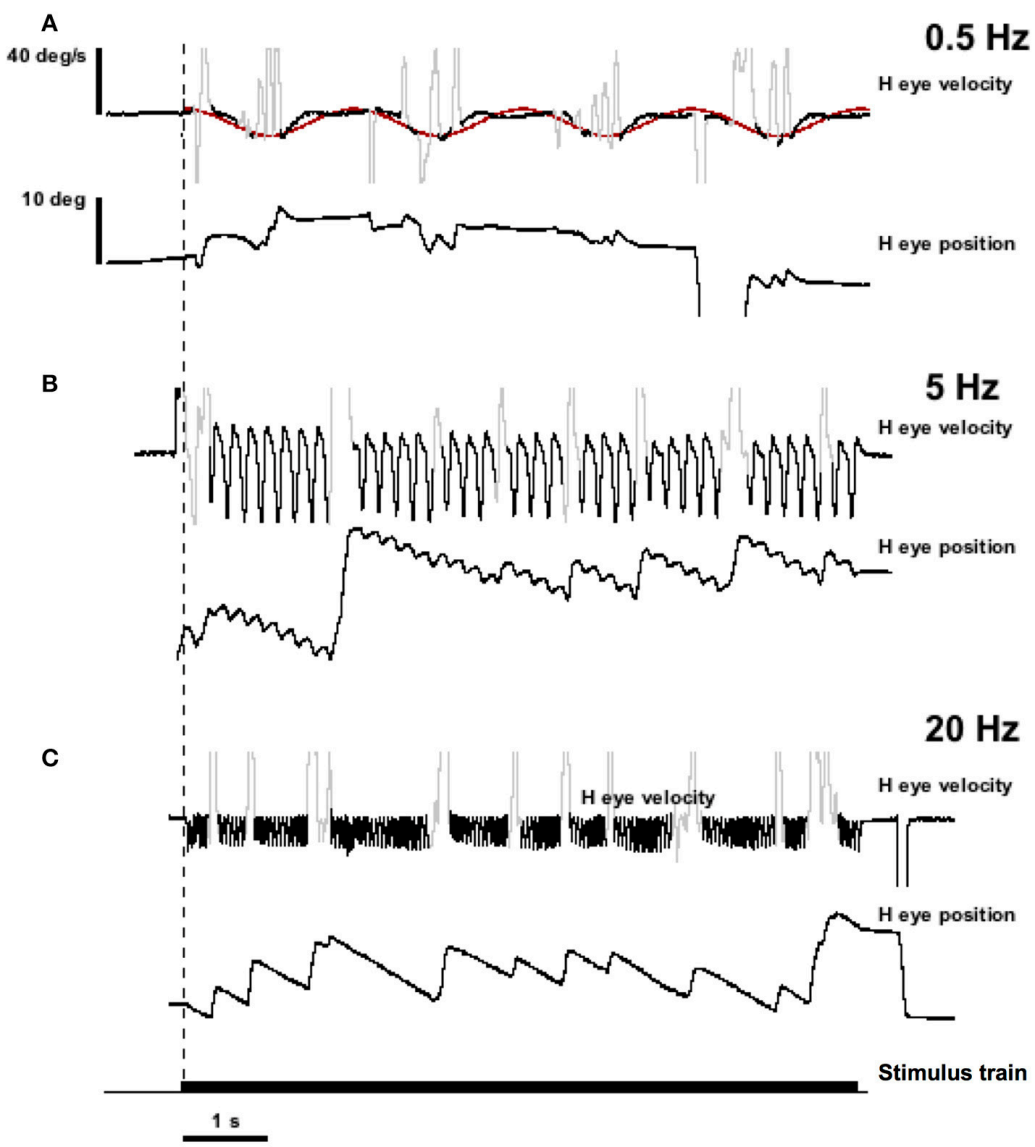
Eye position and velocity resulting from trains of biphasic pulses that are sinusoidally modulated in current amplitude (AM) at different frequencies. Data is from the same animals as the representative traces in Figure 2 (MC6). The pulse frequency is 300 pps. Current amplitude is modulated to up 125 μA maximum. **(A)** Sinusoidal modulation at 0.5 Hz. **(B)** Sinusoidal modulation at 5.0 Hz. **(C)** Sinusoidal modulation at 20 Hz. Traces from top to bottom for each panel are slow phase eye velocity (black) and saccadic velocity (gray), eye position (black). The bottom trace of the figure is the stimulus train. The dashed vertical line indicates the onset of stimulation. The red trace in **(A)** is a sine wave fit to the velocity data.

Figure 4 shows the result of stimulation in the lateral canal of monkey M4 (MC6) at 300 pps with current amplitudes varying sinusoidally between 50 and 125 μA. This is the same canal that was shown in Figure 2A. Horizontal eye movements resulting from three frequencies of sinusiodally modulated electrical stimulation are displayed, as is the sinusoidal fit at the lowest modulation frequency (0.5 Hz). What can be seen immediately from the figure is that the velocity amplitude of the sinusoidally varying slow phase eye velocities is not constant between the three frequencies. At 0.5 Hz in Figure 4A, there is a modest eye velocity that is elicited. At 5.0 Hz (Figure 4B), the sinusoidal eye velocity amplitude is much higher. At 20 Hz (Figure 4C), the velocity amplitude is reduced to levels comparable to the velocity amplitude observed at the 0.5 Hz stimulation. This figure suggests that slow phase eye velocity amplitude elicited by a vestibular prosthesis during time varying stimulation is not constant across frequency. Furthermore, comparison of Figures 2, 3 with Figure 4 suggests that the eye velocities predicted by constant parameter trains do not match the eye velocities observed during time varying stimulus trains, at least for current amplitude modulated (AM) stimulation.

**Figure 5:**
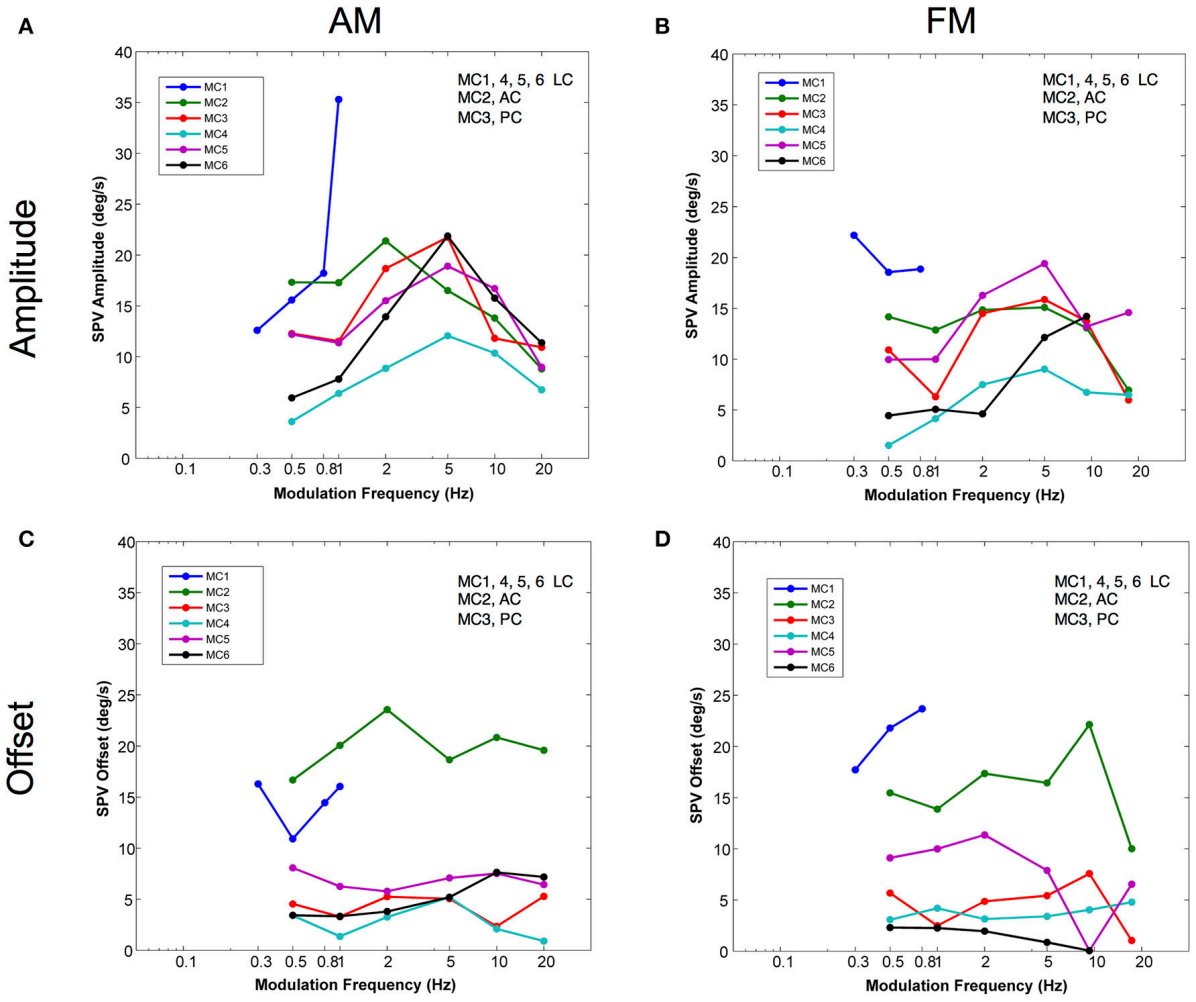
Amplitudes and offsets for sinusoidal fits to the velocity of the eye movements elicited by AM and FM modulated trains of biphasic pulse stimuli at different modulation frequencies. **(A)** Slow phase velocity amplitude vs. AM modulation frequency for 6 canals. **(B)** Slow phase velocity amplitude vs. FM modulation frequency for 6 canals. **(C)** Slow phase velocity offset vs. AM modulation frequency for 6 canals. **(D)** Slow phase velocity offset vs. FM modulation frequency for 6 canals. Positive offsets indicate a shift toward on direction slow phase velocity. Each point represents the average of *n* ≅ 10 cycles. Each line represents data from a separate canal. Canal orientation is indicated as LC (lateral canal), AC (anterior canal), or PC (posterior canal).

To evaluate this phenomenon more rigorously, we calculated the slow phase eye velocity amplitude and offset of sinusoidal fits to eye velocities elicited by current amplitude modulated (AM) and pulse frequency modulated (FM) stimulation across a range of modulation frequencies in 6 canals in 3 monkeys. For the lateral canals, the horizontal component of the eye movements was analyzed. For the vertical canals, the vertical component was analyzed. Four of the canals were lateral canals (MC1, MC4, MC5, and MC6), one canal was a posterior canal (MC3), and one canal was an anterior canal (MC2). The results of this analysis are shown in Figure 5.

For amplitude modulated stimulation, Figure 5A, sinusoidal stimulation of all canals produced an increase in elicited slow phase velocity amplitude with increasing frequency across at least part of the range from 0.5 to 2.0 Hz. Four canals showed a peak amplitude for stimulation at a modulation frequency of 5.0 Hz (MC3, MC4, MC5, MC6). One canal (MC2) showed a peak at 2.0 Hz, and one canal (MC1) could not be modulated at stimulation frequencies above 1.0 Hz, but showed increasing velocity amplitude to that point. Above their peak velocity frequency, all canals with data showed consistent decreases in slow phase velocity amplitude with further increases in frequency. These data show that for AM modulated stimulation trains, all canals show response dynamics with frequency, and most show peak slow phase velocities between 2.0 and 10.0 Hz; i.e., at the 5.0 Hz frequency that was tested.

Current amplitude modulation presumably works through a mechanism that is highly aphysiologic. With increasing current, the electrical stimulation is likely to recruit more afferents to firing, providing more input to the central nervous system. We hypothesized that pulse frequency modulated (FM) electrical stimulation, which is perhaps a better analog of natural afferent activation, might produce different dynamics. To evaluate this, we examined the response to sinusoidal FM electrical stimulation of the same canals stimulated in Figure 5A. Figure 5B shows that very similar relationships exist between modulation frequency and slow phase eye velocity amplitude for FM and AM stimulation. However, for individual canals the responses are not identical. As with AM stimulation, 4 canals showed a peak amplitude for stimulation at a modulation frequency of 5.0 Hz (MC2, MC3, MC4, MC5). One canal (MC6) showed a peak at 10 Hz, but was not tested at higher frequencies. One canal (MC1) had its highest slow phase amplitudes at 0.30 Hz, but was only tested across a limited range of frequencies. Therefore, even the more physiologic FM pulse trains showed significant dynamics with respect to modulation frequency.

We were also interested in the relationship between time varying pulse trains and the 2 s constant parameter pulse trains that we used to map each stimulation site of our device. We observed very little slow phase velocity drift during the rest periods between 2 s stimulation trials in our stimulated canals. This means that electrical stimulation with our device produced unidirectional eye velocity in our 2 s trials. Figures 5C,D show that in fact the eye did move bidirectionally during sinusoidally AM or FM modulated electrical stimulation, respectively. In these figures, an offset of 0 would indicate a perfectly symmetric slow phase velocity. Positive offsets were associated with more slow phase velocity in the on direction (leftward - LC, upward - AC, or downward - PC). All of the offsets were positive, but they failed to match the velocities predicted from the velocity amplitude shown in Figures 5A,B, indicating mostly asymmetric velocity biased toward on direction eye movement for 4 of 6 canals (MC3, MC4, MC5, and MC6), but with significant eye velocity in the off direction as well. The other two canals (MC1 and MC2) had very large offsets indicating largely unilateral velocity across most modulation frequencies.

**Figure 6:**
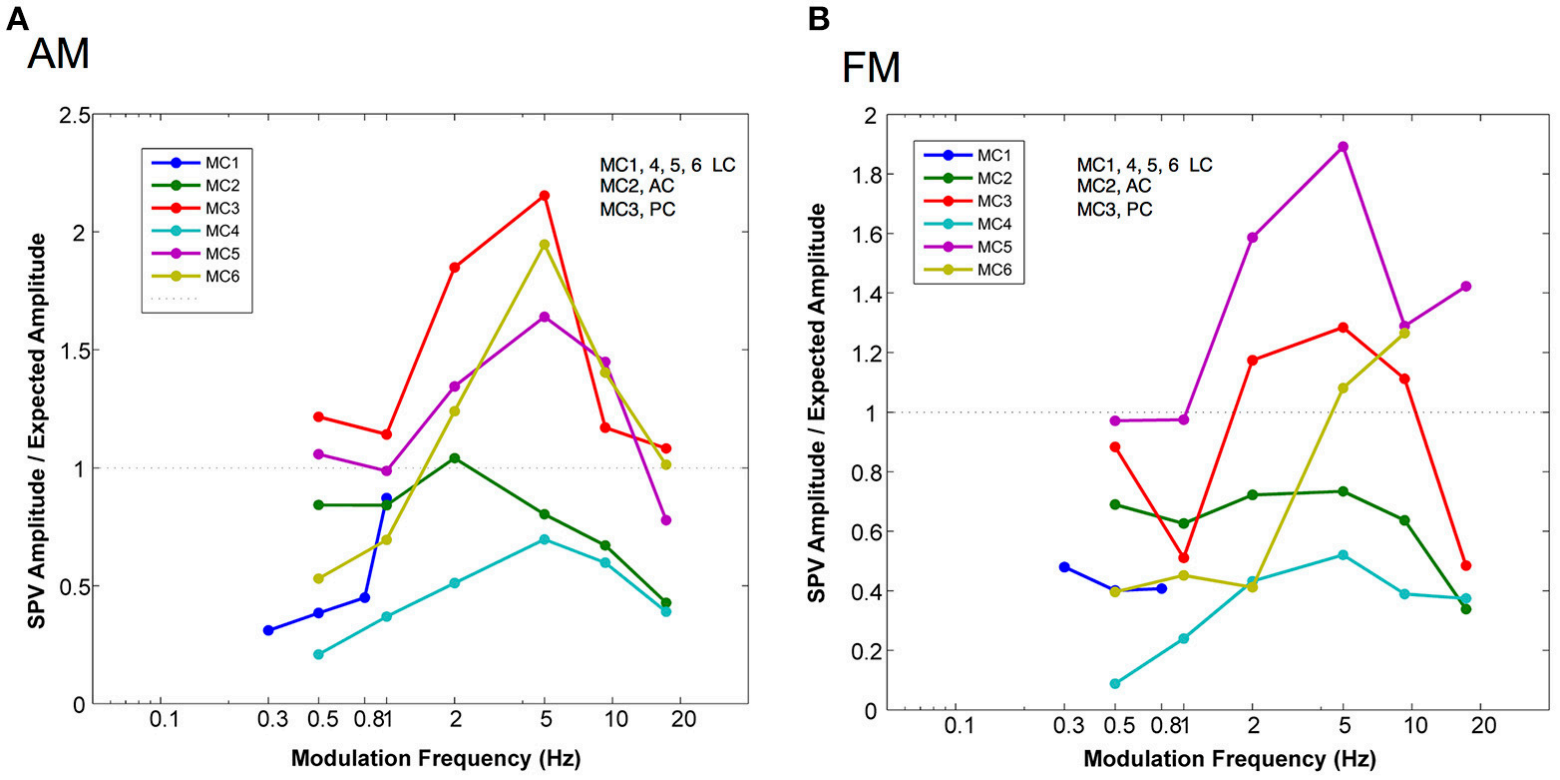
Normalized slow phase amplitude for sinusoidal fits to the velocity of eye movements elicited by AM and FM modulated trains of biphasic pulse stimuli at different modulation frequencies. The traces are normalized by dividing the observed slow phase velocity by the velocities predicted (expected) by the presentation of 2 s constant parameter trains at the same current amplitudes and pulse frequencies as the maximum and minimum values used in the sinusoidally modulated stimuli. **(A)** Normalized slow phase velocity amplitude vs. AM modulation frequency. **(B)** Normalized slow phase velocity amplitude vs. FM modulation frequency. Dotted horizontal lines in both panels indicate a ratio (observed slow phase velocity/expected slow phase velocity) of 1.0. Canal orientation is indicated as LC (lateral canal), AC (anterior canal), or PC (posterior canal).

Also, we were interested in the relationship of the slow phase velocities elicited from 2 s constant parameter stimulation and those elicited by longer trains of time varying stimulation. Recall that the electrical stimulation parameters were adjusted so that the maximum current amplitude and pulse rate of stimulation and the minimum current amplitude and pulse rate of stimulation matched the parameters of 2 s stimulations performed contemporaneously in each canal. Therefore, we hypothesized that we could predict the slow phase velocity amplitudes that we would observe if the electrical stimulation was providing a velocity input, fully characterized by the 2 s stimulation to the central nervous system. In Figure 6 we evaluated this hypothesis by calculating the ratio of the observed slow phase velocity amplitudes to the predicted amplitudes (essentially a gain) for AM and FM stimulation. Figure 6A shows that the low AM frequency amplitudes were below those predicted by the constant parameter stimulation for 4 of 6 canals (MC4, MC1, MC6, MC2), roughly equivalent for one (MC4), and higher for one (MC3). At the peak of the amplitude vs. frequency relationship, 3 of 5 canals (MC5, MC6, and MC3) showed responses were well above the predicted response, one was roughly equivalent (MC2), and one was lower (MC4). At the highest frequency, 3 were lower (MC4, MC5, and MC2), and two were roughly equivalent (MC6 and MC3).

For FM modulation, there were similar differences. At low FM frequency, velocity amplitudes were below those predicted by the constant parameter stimulation for 5 of 6 canals (MC1, MC2, MC3, MC4, and MC6), roughly equivalent for one (MC5), and higher for one (MC3). At the peak of the velocity amplitude vs. modulation frequency relationship, 3 of 5 canals (MC5, MC6, and MC3) showed responses were well above the predicted response, and 2 canals showed lower velocities (MC2 and MC4). At the highest frequency, 3 were lower (MC4, MC5, and MC3), and 2 were higher (MC6 and MC34). Therefore, for both AM and FM stimulation, the response amplitude did not match a simple velocity input model as predicted by the 2 s stimulation.

**Figure 7:**
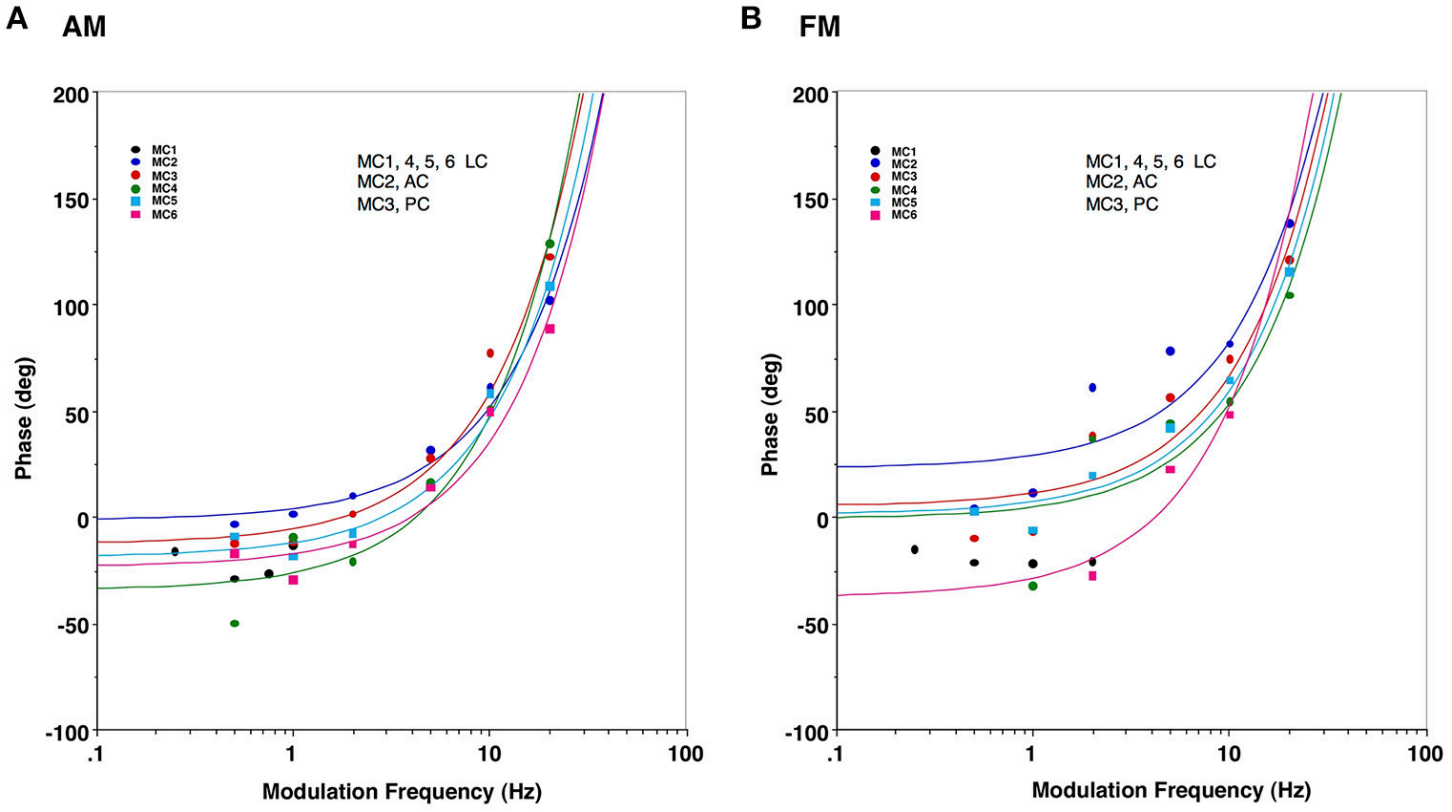
Response phase of slow phase eye movements elicited by AM and FM modulated trains of biphasic pulse stimuli at different modulation frequencies in 6 canals. **(A)** Eye velocity phase vs. AM modulation frequency. **(B)** Eye velocity phase vs. FM modulation frequency. Lines in both panels indicate a linear fit of the phase vs. frequency for a specific canal (represented by upward sloping curves in the log linear plot), with colors matching the corresponding average data points for that canal. Canal orientation is indicated as LC (lateral canal), AC (anterior canal), or PC (posterior canal).

Finally, we predicted that since we bypassed the peripheral vestibular apparatus during our electric stimulation experiments, we would have a relatively constant latency of response across frequencies of stimulation, which would result in a linear phase relationship between our stimulus and the observed behavior (the slope of which would be the group delay). To evaluate this, Figure 7 plots the phase of the response for stimulation of each semicircular canal during AM (Figure 7A) and FM (Figure 7B) stimulation. Since these data are plotted in a log linear plot, the linear relationships between phase and frequency are represented as upward curving lines. The data in Figure 7 indicate that all canals showed fairly comparable linear relationships between phase and frequency across the modulation frequencies studied. The group delays were 19 ± 4 ms for AM modulation and 20 ± 3 ms for FM modulation.

### Experiment 3 (brief constant parameter stimulation at different starting eye positions)

The vestibulo-ocular reflex stabilizes gaze position in space. To do so, it must function not only when the eye is in primary orbital position (straight ahead) but also when the eye is eccentric in the orbit. To accomplish this, the central nervous system must adjust the drive to the extra-ocular muscles to compensate for orbital mechanics, which change with eye position. To evaluate whether this context dependent adjustment takes place during electrical vestibular neurostimulation, we repeated the observations of experiment 1 but with two modifications. First, we reduced the duration of stimulation to restrict the observation of eye movement to single slow phases. Second, we initiated the stimulation when the eye was in secondary orbital positions along either the horizontal or vertical meridian. We then plotted the electrically elicited slow phase eye velocity in response to brief 50 ms, 250 pps constant current amplitude stimulation in monkeys M3 and M4.

**Figure 8:**
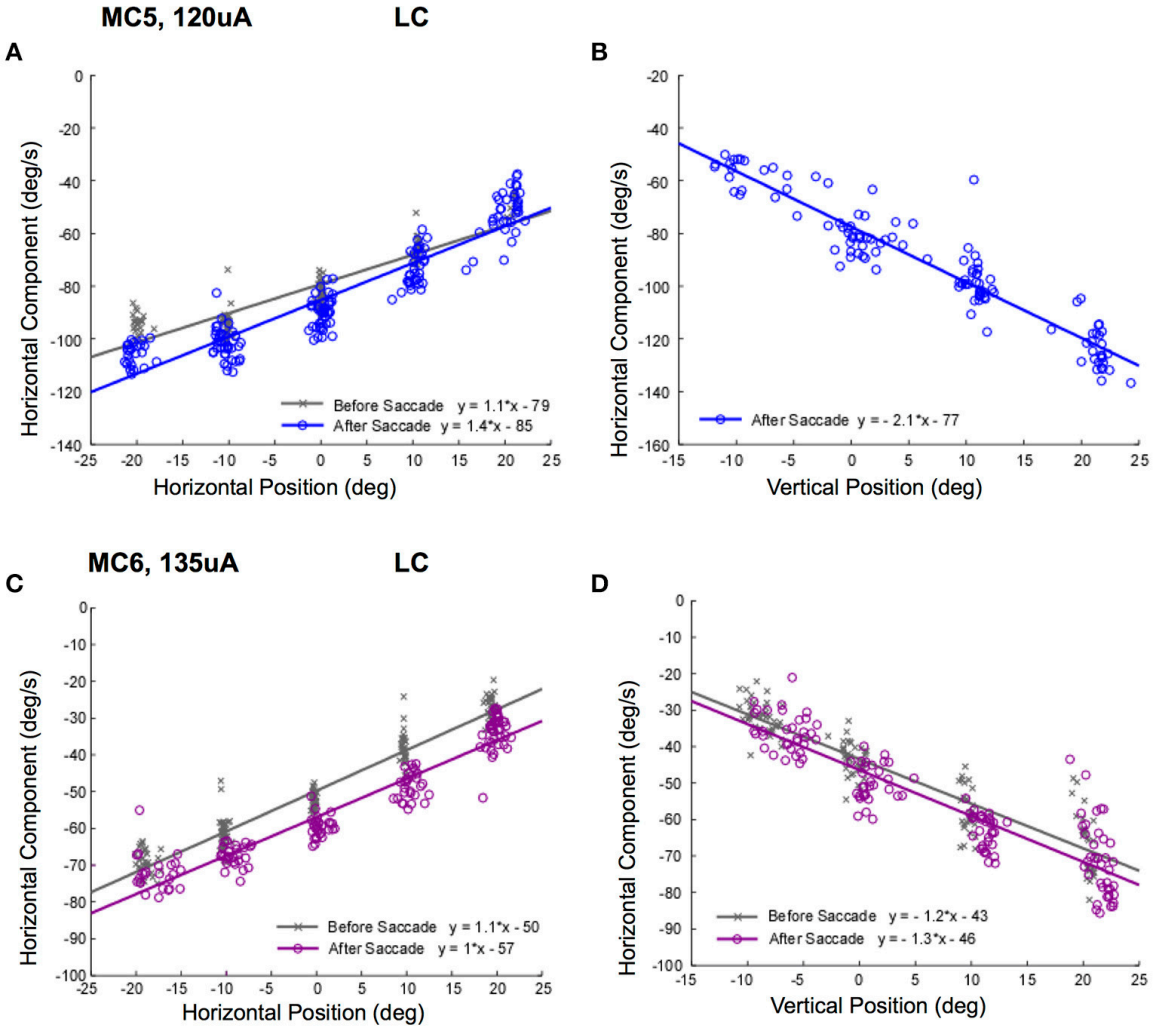
Slow phase eye velocity vs. eye starting position for right lateral canal stimulation. Fifty milliseconds trains of 250 pps biphasic pulses were presented before or after saccades to targets, which were subsequently extinguished for 500 ms during and after stimulation. Data from 2 lateral canals is presented. **(A)** Horizontal component slow phase velocity vs. horizontal starting position for MC5 at 120 μA current amplitude. **(B)** Horizontal component slow phase velocity vs. vertical starting position for MC5 at 120 μA current amplitude. **(C)** Horizontal component slow phase velocity vs. horizontal starting position for MC6 at 135 μA current amplitude. **(D)** Horizontal component slow phase velocity vs. vertical starting position for MC6 at 135 μA. current amplitude. For the upper panels, blue (o) symbols are data for stimulation after the targeting saccade, and gray (x) symbols are data for stimulation before the targeting saccade. For the lower panels, purple (o) symbols are data for stimulation after the targeting saccade, and gray (x) symbols are data for stimulation before the targeting saccade. Negative velocities and positions are leftward or downward. Canal orientation is indicated as LC (lateral canal).

Figure 8 shows the slow phase velocity in the primary horizontal component of the elicited movement for 2 lateral canals (MC5 and MC6) at 120 and 135 μA, respectively. Since we were rewarding the monkeys for tracking a moving target, we compared responses before and after a targeting saccade to disambiguate the response velocities and the saccadic tracking paradigm. For one condition, starting positions along the vertical meridian for MC5, it was not possible to do this, and so only post-saccade data is shown. Figures 8A,C show the relationship between horizontal show phase velocity and horizontal eye position before and after the saccades. There was a clear relationship observed between eye position, and elicited eye velocity. This was true independent of the temporal relationship of stimulation onset to the saccade aligning the eye on the starting position. For right lateral canal stimulation, leftward slow phase eye velocities recorded in the left eye in response to stimulation of both canals were higher as the starting position was moved to the left. These differences were large and the slope of the relationship was significantly different from 0 for all fits (*P* ≤ 0.05). Therefore, the further in the slow phase velocity direction that the eye started, the higher the observed velocity.

The relationship between elicited slow phase velocity and eye starting position also held for vertical eye positions. As the eye starting position moved from down eye positions to up eye positions, the elicited leftward velocity increased significantly (Figures 8B,D). Again, these differences were large and the slope of the relationship was significantly different from 0 for all fits (*P* ≤ 0.05).

**Figure 9:**
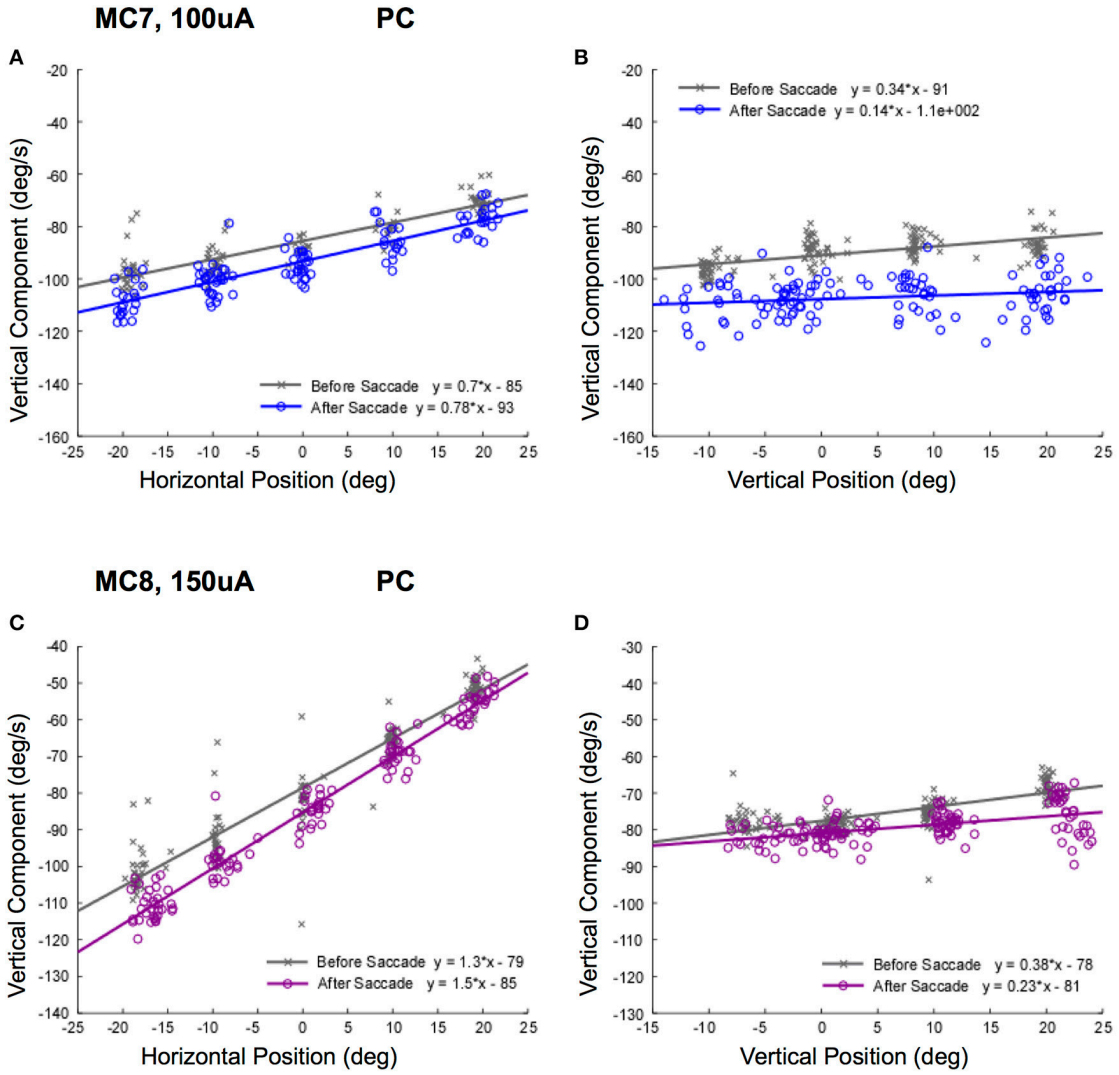
Slow phase eye velocity vs. eye starting position for right posterior canal stimulation. Fifty milliseconds trains of 250 pps biphasic pulses were presented before or after saccades to targets, which were subsequently extinguished for 500 ms during and after stimulation. Data from 2 posterior canals is presented. **(A)** Vertical component slow phase velocity vs. horizontal starting position for MC7 at 100 μA current amplitude. **(B)** Vertical component slow phase velocity vs. vertical starting position for MC7 at 100 μA current amplitude. **(C)** Vertical component slow phase velocity vs. horizontal starting position for MC8 at 150 μA current amplitude. **(D)** Vertical component slow phase velocity vs. vertical starting position for MC8 at 150 μA current amplitude. For the upper panels, blue (o) symbols are data for stimulation after the targeting saccade, and gray (x) symbols are data for stimulation before the targeting saccade. For the lower panels, purple (o) symbols are data for stimulation after the targeting saccade, and gray (x) symbols are data for stimulation before the targeting saccade. Negative velocities and positions are leftward or downward. Canal orientation is indicated as PC (posterior canal).

This relationship was striking for right lateral canal stimulation, but we only recorded movements in the left eye. It is possible that there was a superimposed horizontal divergence movement that contributed to the response. In order to control for this, we examined the relationship vertical slow phase eye velocity and left eye position for right posterior canal stimulation. The data resulting from stimulations of the posterior canals of monkeys M3 (100 μA) and M4 (150 μA) at different orbital eye starting positions are shown in Figure 9. Again, there was a clear relationship between observed horizontal eye position, and elicited vertical eye velocity (Figures 9A,C). This also was true independent of the temporal relationship of stimulation onset to the saccade aligning the eye on the starting position. For right posterior canal stimulation, downward slow phase eye velocities recorded in the left eye in response to stimulation of both canals were higher as the staring position was moved to the left. The slope of the relationship was significantly different from 0 for all fits (*P* ≤ 0.05).

The relationship between elicited vertical slow phase velocity and eye starting position had smaller slopes for vertical eye positions. As can be seen in Figures 9B,D, as the eye starting position moved from up eye positions to down eye positions, the elicited leftward velocity increased slightly. These differences were very small, but the slopes of the relationships were significantly different from 0 for all fits (*P* ≤ 0.05).

Taken together, the data of Figures 8, 9 suggest that the typical compensation for orbital mechanics may not be present during electrical neurostimulation, since the same stimulus, presumably coding for the same eye velocity, produced different response velocities in different starting orbital locations.

**Figure 10:**
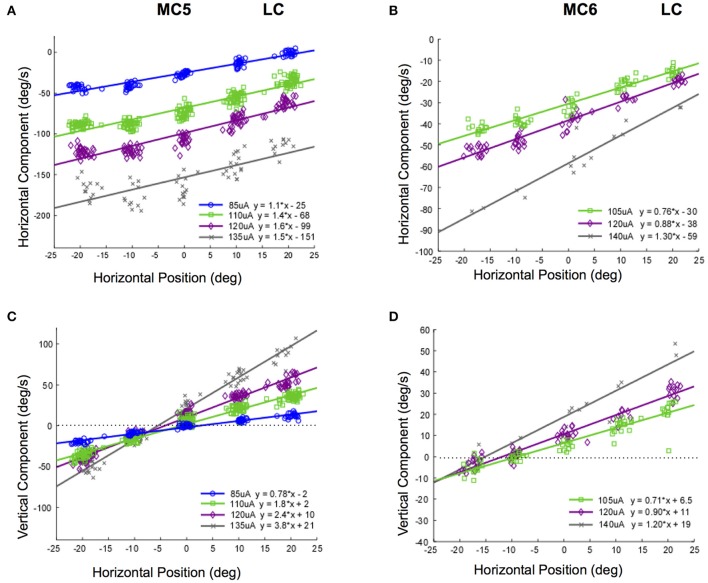
Slow phase eye velocity vs. eye starting position for right lateral canal stimulation at different current amplitudes. Fifty milliseconds trains of 250 pps biphasic pulses were presented after saccades to targets, which were subsequently extinguished for 500 ms during and after stimulation. Data from 2 lateral canals is presented. **(A)** Horizontal component slow phase velocity vs. horizontal starting position for MC5 at different current amplitudes. **(B)** Horizontal component slow phase velocity vs. horizontal starting position for MC6 at different current amplitudes. **(C)** Vertical component slow phase velocity vs. horizontal starting position for MC5 at different current amplitudes. **(D)** Vertical component slow phase velocity vs. horizontal starting position for MC6 at different current amplitudes. For all panels, different symbols represent different current amplitudes. For **(C,D)**, horizontal lines indicate 0 velocity. Negative velocities and positions are leftward or downward. Canal orientation is indicated as LC (lateral canal).

It is possible that the response velocity changes observed in Figures 8, 9 were only idiosyncratically present at higher stimulus currents. In addition, these changes in slow phase velocity magnitude may have been related to changes in the direction of the slow phase eye velocity. To address this issue we performed stimulations at 250 pps at different stimulation current amplitudes in the 4 canals in monkeys M3 and M4. Figure 10 shows the data for such stimulation in the lateral canals (MC5 and MC6), displaying both the horizontal and vertical components of the resulting eye velocity. It is clear from the data of Figure 10 that increasing the current amplitude of the stimulation does increase the observed slow phase velocity of the elicited eye movements. However, a strong relationship between starting horizontal eye position and slow phase eye velocity remains. In addition, the direction of the eye movements does change. This is most dramatically seen in the horizontal and vertical eye velocities elicited in canal MC5, where the magnitude of the horizontal slow phase velocity increases with stimulation current from 85 to 135 μA, but the slope of the relationship between horizontal eye velocity and horizontal starting position remains relatively unchanged (Figure 10A). However, both the slope and the direction of the vertical component change with horizontal starting position and current amplitude (Figure 10C). Comparable, but less dramatic changes in eye velocity magnitude and direction with eye starting position at different current amplitudes occurs in canal MC6 (Figures 10B,D).

**Figure 11:**
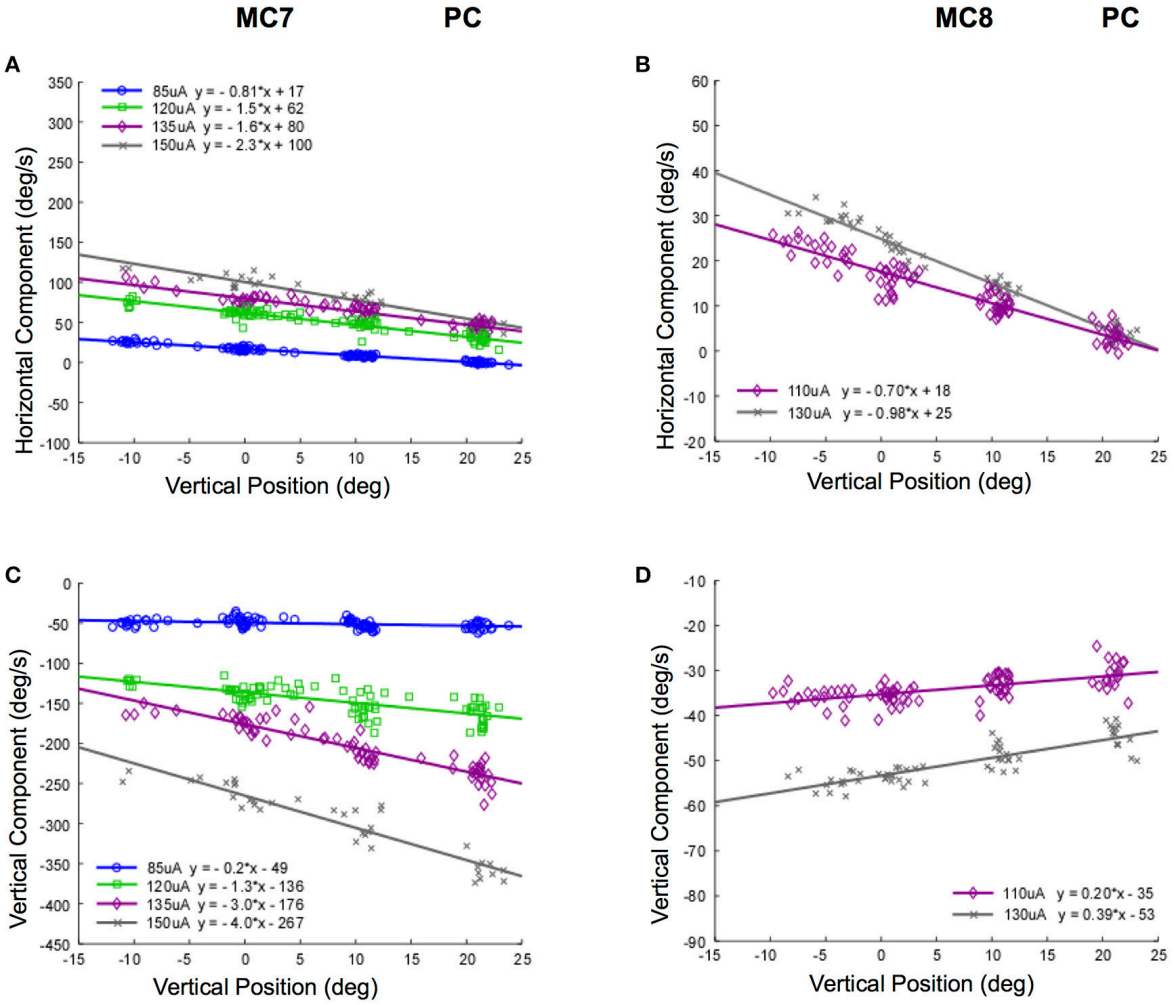
Slow phase eye velocity vs. eye starting position for right posterior canal stimulation at different current amplitudes. Fifty milliseconds trains of 250 pps biphasic pulses were presented before or after saccades to targets, which were subsequently extinguished for 500 ms during and after stimulation. Data from 2 posterior canals is presented. **(A)** Horizontal component slow phase velocity vs. vertical starting position for MC7 at different current amplitudes. **(B)** Horizontal component slow phase velocity vs. vertical starting position for MC8 at different current amplitudes. **(C)** Vertical component slow phase velocity vs. vertical starting position for MC7 at different current amplitudes. **(D)** Vertical component slow phase velocity vs. vertical starting position for MC8 at different current amplitudes. For all panels, different symbols represent different current amplitudes. Negative velocities and positions are leftward or downward. Canal orientation is indicated as PC (posterior canal).

Different changes in the relationship between slow phase eye velocity and eye starting position with current amplitude occur for stimulation of the posterior canals in monkeys M3 and M4. Figure 11 shows these relationships for canals MC7 and MC8. Figures 11A,C show the horizontal and vertical velocity vs. vertical eye position relationships for stimulation current amplitudes of 85–150 μA in MC7. As current increases, there is an increase in the magnitude of the vertical and horizontal slow phase velocity component of the resulting eye movement, but also an increase in the slope of the relationship between eye velocity and eye position. While there is no change in the direction of the observed vertical eye movement components, the ratio of the vertical slope to the horizontal slope changes from 0.25 to 1.7 as currents progress from 85 to 150 μA. This indicates a clear change in the direction of the observed eye movements with eye position at different current amplitudes. There is less data for these relationships in canal MC8, Figures 11B,D. In this case, the ratio of the vertical component slope to the horizontal component slope in the relationship between component velocity and vertical eye position changes from 0.40 to 0.28 as currents progress across a rather limited range of current amplitudes from 110 to 130 μA. Although the changes in slope are significant, the magnitude of the change is relatively small.

Taken together, the data of Figures 10, 11 suggest that not only is there a relationship between electrically elicited slow phase eye velocity magnitude and starting eye position, but there is a relationship between electrically elicited slow phase eye velocity direction and eye starting position. Furthermore, this relationship changes with changes in current amplitude. These changes are somewhat unexpected, and suggest that the mechanisms that compensate for orbital dynamics in the natural vestibulo-ocular reflex are not fully compensatory during electrical vestibular neurostimulation in the dark.

### Experiment 4 (constant parameter stimulation in different head orientations)

The angular VOR must be capable of stabilizing gaze position in space during head rotation independent of the orientation of the head in space. Ordinarily this is accomplished through a combination of convergent canal and otolith signals. It is difficult to know precisely how these signals combine in real world rotations, because there is both a rotational stimulus to the canals and a changing gravitational vector to the otolith organs. However, the vestibular neurostimulator gave us an opportunity to examine this directly. Our hypothesis was that the response to vestibular neurostimulation would be identical in different static head orientations, because there would be no corresponding change in otolith input during the stimulation. This would be interpreted by the central nervous system as a pure rotational input, and the electrically elicited canal input alone would drive eye velocity.

To test this hypothesis, we examined the slow phase nystagmus eye velocity response to 2 s trains of constant current amplitude and constant pulse frequency biphasic pulse stimulation in different head orientations; i.e., upright in the stereotaxic plane, 45 degree pitch nose down or nose up, or 45 degree tilt roll left or right. The stimulation was initiated with the eye in primary position, and the static head orientation was controlled by pseudorandom changes in en-block monkey orientation by activation of the 3D rotator in the dark.

**Figure 12:**
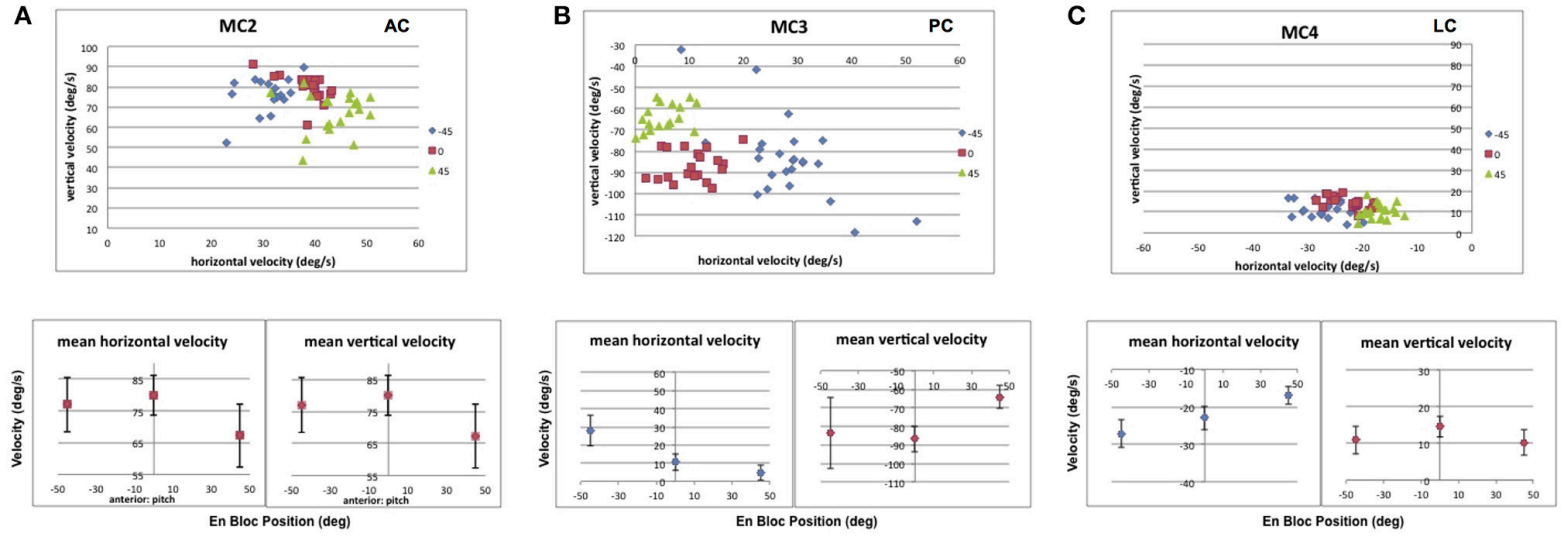
Average horizontal and vertical slow phase velocity elicited by 2 s constant current amplitude and constant pulse frequency stimulation in 3 canals during static pitch tilt in 3 orientations, upright in the stereotaxic plane (0°), 45° pitch nose down (45°), and 45° pitch nose up (−45°). Upper panels display horizontal and vertical slow phase velocity components for trials within blocks. Different symbols represent different orientations. Lower panels display means ± SD for slow phase velocity vs. orientation. **(A)** Data for anterior canal MC2. **(B)** Data for posterior canal MC3. **(C)** Data for lateral canal MC4. Canal orientation is indicated as LC (lateral canal), AC (anterior canal), or PC (posterior canal).

Figure 12 shows the result of static pitch tilt on the recorded eye velocities elicited from stimulation of the 3 semicircular canals in monkey M2 (MC2, anterior; MC3, posterior; MC4, lateral). In Figure 12A, the data shows that for stimulation of right anterior canal MC2, static nose down pitch tilt produces a statistically significant reduction in the vertical and horizontal slow phase velocity of the electrically elicited eye movement from that observed during upright orientation. In static pitch tilt nose up, neither component is reduced. For right posterior canal MC3 stimulation, Figure 12B, there is a statistically significant reduction in the vertical component and horizontal components of the elicited slow phase eye velocity in static pitch tilt nose down relative to upright, but an increase only in the horizontal component in static pitch tilt nose up. For lateral canal MC4 stimulation, in Figure 10C, there is again a statistically significant decrease in mean horizontal and vertical slow phase velocity during static pitch tilt nose down, but an increase in horizontal slow phase velocity and a decrease in the vertical slow phase velocity during static pitch tilt nose up. Therefore, for pitch tilt responses, our hypothesis was incorrect. Pitch tilt nose down always reduced the slow phase velocity elicited by electrical stimulation. Pitch tilt nose up produced variable results across canals and component directions.

**Figure 13:**
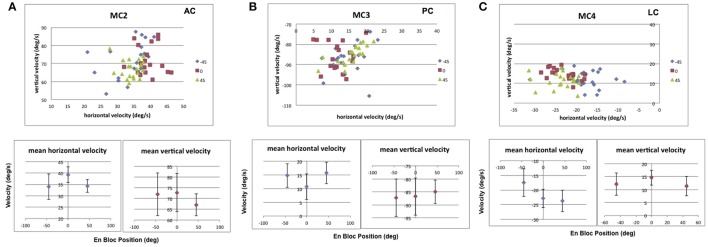
Average horizontal and vertical slow phase velocity elicited by 2 s constant current amplitude and constant pulse frequency stimulation in 3 canals during static roll tilt in 3 orientations, upright in the stereotaxic plane (0°), 45° roll right (45°), and 45° roll left (−45°). Upper panels display horizontal and vertical slow phase velocity components for trials within blocks. Different symbols represent different orientations. Lower panels display means ± SD for slow phase velocity vs. orientation. **(A)** Data for anterior canal MC2. **(B)** Data for posterior canal MC3. **(C)** Data for lateral canal MC4. Canal orientation is indicated as LC (lateral canal), AC (anterior canal), or PC (posterior canal).

For roll tilt orientation, there were also changes in electrically elicited slow phase velocity depending on tilt orientation either toward the stimulated ear (static roll tilt right) or away from the stimulated ear (static roll tilt left). Figure 13 shows the result of static roll tilt on the recorded eye velocities elicited from stimulation of the 3 semicircular canals discussed above (MC2, anterior; MC3, posterior; MC4, lateral). In Figure 13A, the data shows that for stimulation of right anterior canal MC2, roll tilt toward that canal produces a statistically significant reduction in the vertical and horizontal slow phase velocity of the electrically elicited eye movement from that observed during upright orientation. In roll tilt left, only the horizontal component is reduced. For right posterior canal MC3 stimulation, Figure 13B, there is a statistically significant increase in the horizontal component and no change in the vertical components of the elicited slow phase eye velocity in static roll tilt toward or away from the stimulated canal relative to upright. For lateral canal MC4 stimulation, in Figure 11C, there is again a statistically significant decrease in mean vertical slow phase velocity and no change in horizontal velocity during static head tilt toward the stimulated canal, but a significant decrease in horizontal slow phase velocity and no change in the vertical slow phase velocity during roll tilt left. Therefore, for roll tilt responses, our hypothesis was again incorrect. Roll tilt toward or away from the stimulated canal always reduced at least one component of the observed slow phase velocity elicited by electrical stimulation of the 3 semicircular canals.

## Discussion

In this paper we examined the relationships between slow phase eye movement velocity and electrical stimulation parameters during eye movements elicited by biphasic pulse electrical stimulation with a unilateral vestibular neurostimulator in a range of different contexts. The purpose was to evaluate the behavioral results of such stimulation without making a priori assumptions about the types of transformations that should exist between the electrical stimulation and the behavioral measures that define the efficacy of that stimulation.

From Experiment 1, we observed that short constant parameter pulse trains seem to provide, to a first approximation, constant velocity input to the vestibular system; i.e., they elicit constant velocity slow phase nystagmus. Changes in current amplitude and in pulse frequency produce parametric changes in the observed slow phase velocities elicited by the stimulus trains. Unilateral stimulation produces unilaterally directed slow phase velocities. This is consistent with previous reports from our laboratory and others in monkeys and humans (e.g., Cohen and Suzuki, 1963; Cohen et al., 1964; Suzuki and Cohen, 1964; Wall et al., 2007; Guyot et al., 2011b; Lewis et al., 2013; Phillips et al., 2014, 2015, 2016). Experiment 2 revealed that AM or FM sinusoidally modulated trains of biphasic pulses produced slow phase velocity amplitudes that were not well predicted by the brief constant parameter stimulation of Experiment 1. At low and very high frequencies of modulation, on average the constant parameter stimulation overestimated the resulting sinusoidal velocity amplitudes, and at moderate modulation frequencies, on average the constant parameter stimulation underestimated the observed sinusoidal velocity amplitudes. This feature of the response was true for both pulse frequency modulated (FM) stimulation and current amplitude modulated (AM) stimulation, despite the likely differences in neural mechanism between these two different stimulation strategies.

Changes in eye velocity gain during en-bock rotation with rotation modulated vestibular neurostimulation at different frequencies have been reported in human subjects with vestibular loss (Perez Fornos et al., 2014; van de Berg et al., 2015). These experiments were performed with amplitude modulated biphasic pulse stimulation over smaller modulation frequency ranges; i.e., 0.5–2.0 Hz modulation and 0.1–2.0 Hz modulation, respectively. One animal study (Dai et al., 2011a) showed similar findings for frequency modulated biphasic pulse stimulation over the frequency range from 0.2 to 5.0 Hz. Most animal studies of neuroprosthetic stimulation are performed across even more restricted frequency ranges, or at a single modulation frequency (Gong and Merfeld, 2000, 2002; Lewis et al., 2001, 2002, 2010, 2013; Della Santina et al., 2005, 2007; Merfeld et al., 2007; Gong et al., 2008; Fridman et al., 2010; Chiang et al., 2011; Dai et al., 2011b,c, 2013; Davidovics et al., 2011, 2013; Phillips et al., 2011; Sun et al., 2011; Nie et al., 2013). In both studies where the extended modulation frequency range was studied, the authors observed similar responses to those observed here; i.e., a general increase in gain with modulation frequency. However, in the current study, as the modulation frequencies were increased beyond 5.0 Hz, the gains actually decrease as modulation frequency continues to increase. This result is inconsistent with the responses to natural rotation that have been observed in previous experiments with intact monkeys (e.g., Ramachandran and Lisberger, 2005).

Rotational models of afferent input do not really explain this result. Studies in monkeys have shown that the sensitivity of vestibular afferents increases with increasing rotation frequency at the higher frequencies studied here (e.g., Ramachandran and Lisberger, 2006). While regular neurons show discharge expected from the torsion pendulum model of vestibular end organ mechanics at very low frequencies, irregular neurons do not. However, across the moderately lower frequencies studied here, both afferent types should reflect the dynamics of the cupula; i.e., they should report head velocity to the central nervous system. Therefore, the constant parameter stimulation should closely predict the response to low frequency modulation. Actually, it overestimates the slow phase velocity of the response. At higher frequencies, during normal rotation both afferent types show higher gains with increasing frequency (Schneider and Anderson, 1976; Tomko et al., 1981; Curthoys, 1982; Baird et al., 1988). Above 5.0 Hz, our behavioral results suggest that the gain dramatically decreases as opposed to increasing. Perhaps it is the case that electrical stimulation, by bypassing the normal hair cell transduction mechanism and ionic channels that mediate the natural response, produce an afferent input that fails to increase with frequency. This unexpected afferent input to the CNS produces reduced behavioral output because the vestibular system overall expects more input gain than the electrical stimulation provides.

It is possible that our results could be explained by habituation of the response to electrical stimulation during our recording sessions. Our 2 s stimulation trials were specifically designed to eliminate adaptive changes by reducing the duration of the stimulation so that we could perform accurate longitudinal measurement of stimulation efficacy across many months in animal and human subjects (Phillips et al., 2014, 2015). The sinusoidal modulation trials were, by necessity, of longer duration. However, it should be noted that the modulation frequency of sequential trials of stimulation was pseudorandomly varied, and the same relationships between modulation frequency and slow phase velocity amplitude were observed within and across canals and animals. A second possible explanation for our data is that the high pulse rate stimulation produced polarization of our electrodes, reducing the efficacy of the stimulation. This explanation also seems unlikely given that there was no change in the slow phase velocity amplitude of the response between the beginning and end of a particular trial of stimulation (see Figure 3) and the trials were pseudorandomly presented. Third, the electrical stimulation may have resulted in orthodromic and antidromic activation of vestibular afferents and vestibular efferents. It is difficult to predict what behavioral effects would result from such stimulation.

Finally, our results may be due to the preferential activation of specific vestibular afferent types or stimulation of multiple end organs. The afferent fibers display a range of properties in terms of the regularity of their resting firing rates, their sensitivity to the velocity and acceleration of natural head rotation, their conduction velocities, their central targets, and either their galvanic sensitivity or their sensitivity to vestibular efferent stimulation (Fernández and Goldberg, 1971, 1976; Goldberg and Fernandez, 1971a,b; Goldberg and Fernández, 1977, 1980; Schneider and Anderson, 1976; Yagi et al., 1977; Curthoys, 1982; Goldberg et al., 1982, 1984, 1990; Ezure et al., 1983; Baird et al., 1988; Fernández et al., 1988; Brontë-Stewart and Lisberger, 1994; McCue and Guinan, 1994; Lysakowski et al., 1995; Goldberg, 2000; Marlinski et al., 2004; Sadeghi et al., 2007). For example, since irregular afferents are preferentially activated by galvanic stimulation, we are likely to be disproportionately activating this class of afferent.

The most puzzling feature of the sinusoidal velocity amplitude response is that at moderate frequencies of ~5 Hz there is a sweet spot, where the velocity amplitudes are maximal and are, on average, underestimated by the constant parameter stimulation. In addition, there was another striking finding of the sinusoidal modulation experiments. That was the emergence of bidirectional slow phase eye velocity absent from the constant parameter stimulation experiments but immediately apparent during sinusoidal modulation. In our experiments, sinusoidal stimulation but not constant parameter stimulation produced bidirectional eye velocity. There was always a significant offset toward eye velocity coherent with the on direction of eye movement elicited by the stimulated canal (e.g., leftward for right lateral canal stimulation and downward for posterior canal stimulation). The emergence of this bidirectional response, which had been seen in chronic stimulation experiments (e.g., Lewis et al., 2001, 2002, 2013; Dai et al., 2011a), suggests that modulation *per se* produces the change in direction. These results are not as dramatic as the symmetry seen in the results of van de Berg and colleagues in humans over a more limited frequency range (van de Berg et al., 2015).

Another surprising result was that of Experiment 3, where changes in eye position produced very significant changes in the magnitude and direction of the slow phase velocity response to brief constant parameter stimulation. This was true despite the fact that we controlled for the effects of superimposing a saccadic tracking task on the short duration biphasic pulse electrical stimulation in intermittent darkness. A simple explanation for this might have been that the response was following Alexander's law, except that in fact the changes in eye velocity were directly opposite to those predicted by that rule; i.e., slow phase velocity increased with starting eye position in the direction of the slow phase. Also, this means also that centrifugal movement vs. centripetal movement velocity differences, predicted by the elasticity of the oculomotor plant, fail to explain the changes in the electrically elicited eye velocity because they too are in the wrong direction; i.e., the velocities are higher when the passive elasticity is higher in the opposite direction. One possible explanation for the observations here is that there is a convergence of eye position and vestibular input on secondary vestibular neurons. It is possible that the convergence of an abnormal electrically elicited input to these neurons and a natural eye position input is not appropriately summed to produce a context appropriate behavior. Another possibility is that the agonist motoneurons were in a higher state of activation as the eye starting position moved in the direction of the slow phase velocity. This would allow a stronger transient response to the incoming electrically elicited vestibular input. This hypothesis would explain the primary component of the response, but fails to explain the smaller orthogonal velocity component, and certainly fails to predict the reversal of the orthogonal eye velocity movement direction at some stimulation currents. Rather, it appears that there is an unexpected convergence of inputs during electrical stimulation for which the central nervous system cannot or does not fully compensate during brief electrical stimulation when the eye is in eccentric positions.

Finally, Experiment 4 demonstrated that eye velocities were not constant for 2 s electrical stimulation trains of fixed current amplitude and pulse frequency when the stimulations were performed in different head orientations. This was true for both horizontal canal stimulation and for stimulation of the vertical canals in both pitch and roll tilt. This result might be expected if the central neurons which process the rotational inputs from the canal stimulation were receiving convergent input from the otolith organs. We had predicted that a lack of modulated otolith input would reduce or eliminate the effect of this convergence. With any non-horizontal rotation, there is progressive modulation of otolith input. This was likely not the case, unless our stimulation produced current spread to the otolith organs. On the one hand, this result suggests that there are predictable interactions between otolith and canal inputs in the situation where the canal inputs are provided by biphasic pulse neurostimulation. This is a necessary precondition for successful implementation of a vestibular neuroprosthesis. On the other hand, since the stimulation paradigm produced atypical combinations of canal and otolith input, it is difficult to say what combinations of rotational and tilt or translational signals the central nervous system was extracting from this situation. One possible explanation for the different response to electrical stimulation is that placing the animals in different en-block orientations changed the neck afferent input to secondary vestibular neurons, which then affected the excitability of those neurons to vestibular afferent input. We used en-block rotations specifically to reduce this effect, but since we did not monitor neck EMG, we cannot be certain that this did not occur.

The significance of these experiments is that they have revealed that the vestibular system does not behave in a manner that is entirely predictable from its natural behavior in response to rotational stimuli when you provide a simple fictive rotational stimulus via a vestibular neurostimulator. In the past, investigators have often made logical assumptions about the relationships between the sensory transformations that are known to take place in the natural vestibular system and those that would be needed to successfully implement a vestibular prosthesis. For example, one could use the time constants and velocity and acceleration transformations that are known to exist in the peripheral vestibular system, and implement these in the conversion of a head rotational input to a biphasic pulse train. The underlying assumption of this approach is that the stimulation train would uniformly activate the appropriate afferent populations in a physiological manner, providing a rate code with the logical average weighting of the overall natural modulation of the system. Of course, in detail, this is unrealistic. However, it was and is a logical starting point.

The limitations to this approach are imposed by the nature of the electrical stimulus and the individual afferents. First, afferents are not uniformly represented in terms of their resting rate, their sensitivity to head velocity and acceleration, their size and conduction velocities, their terminal location in the end organ, or their central projections. Indeed, there is a continuum of such properties within the afferent populations that project to any given motor system or central process. Second, the extent to which a given afferent is galvanically sensitive to microstimulation with a vestibular neurostimulator may be strongly correlated to several of these continuous properties. For example, large, irregular, rapidly conducting afferents are the most galvanically sensitive to large DC current. How this maps specifically to small biphasic pulse local stimulation is unknown. Different afferent types are differentially represented in different parts of the end organ. Therefore, local electrical stimulation may drive some but not all of the irregular afferents in combination with less galvanically sensitive but more locally situated regular afferents. Many of the regular and irregular afferents provide an admixture of velocity and acceleration input to the CNS, in part because of the hair cells to which, and synaptic specializations through which, they connect to the transduction apparatus. Therefore, with different stimulation currents we may be activating different populations of afferents, each representing a labeled line from which the nervous system is expecting specific velocity and acceleration information.

Centrally, this confusion between how we actually drive the vestibular system and what the central neurons expect is further complicated by the fact that many vestibular neurons are expecting convergent and complimentary inputs from different afferent types representing inputs from different end organs, often both canal and otolith organs, and information from both ears relayed via commissural inputs from the contralateral nucleus, and parallel pathways through the vestibulo-cerebellum. This means of course, that the vestibular nuclear neurons are expecting inputs that they never receive during electrical vestibular neurostimulation.

Finally, the cerebellum and other higher order centers are constantly adjusting the inputs to the vestibular neurons and the downstream extraocular motoneurons that drive the extraocular muscles responsible for the eye movement behaviors that we use in animals and human subjects as a dependent measure of the efficacy of stimulation. This adaptive processing compensates for changes in the sensory input, but also for the complexities of the motor plant. It is not fully known what specific features of the vestibular input allow full implementation of these central adjustments.

All of these features of the vestibular system must be accommodated, either through careful construction of the electrical stimulus parameters in a vestibular sensory neural prosthesis or through motor learning resulting from continued exposure to the electrically elicited vestibular stimulus. Indeed, it is clear that such motor learning does in fact take place in response to longer-term electrical neurostimulation (Lewis et al., 2001, 2002, 2013; Merfeld et al., 2006; Dai et al., 2011a, 2013; Guyot et al., 2011a). However, it is unknown what the dynamic range and specific operating characteristics of the motor learning are with respect to electrically elicited vestibular stimuli. It is hoped that neural recording and additional adaptation experiments will ultimately provide a more complete understanding of the mechanisms underlying the difference between natural rotational vestibular stimulation and electrical vestibular neurostimulation, as well as insights into the adaptive process required to reconcile these two inputs.

## Author contributions

JP: Oversight of the research project, experimental design, data collection, data analysis, writing the manuscript, and editing the manuscript; LL, AN, and CP: Experimental design, surgical preparation, data collection, data analysis, and editing the manuscript; KN: Experimental design, surgical preparation, data collection and editing the manuscript; JR: Oversight of the research project, experimental design, surgical preparation, and editing the manuscript.

### Conflict of interest statement

JR has been a paid consultant for Cochlear, Ltd., which manufactured and provided the UW/Nucleus vestibular implant. LL, KN, JP, JR and the University of Washington hold intellectual property rights to the device used in this study. The other authors declare that the research was conducted in the absence of any commercial or financial relationships that could be construed as a potential conflict of interest.
